# Instrumented Posterolateral fusion versus instrumented Interbody fusion for degenerative lumbar diseases in uremic patients under hemodialysis

**DOI:** 10.1186/s12891-020-03815-z

**Published:** 2020-12-05

**Authors:** Chia-Ning Ho, Jen-Chung Liao, Wen-Jer Chen

**Affiliations:** Department of Orthopedics Surgery, Bone and Joint Research Center, Chang Gung Memorial Hospital, Chang Gung University, No._5, Fu-Shin Street, Kweishian, Taoyuan, 333 Taiwan

**Keywords:** End stage renal disease (ESRD), Degenerative lumbar diseases, Lumbar posterolateral fusion, Lumbar interbody fusion, Functional outcomes, Complications

## Abstract

**Background:**

Advances in hemodialysis have facilitated longer lifespan and better quality of life for patients with end stage renal disease (ESRD). Symptomatic degenerative lumbar diseases (DLD) becomes more common in patients with ESRD. Posterior instrumented fusion remains popular for spinal stenosis combining instability. Only a few sporadic studies mentioned about surgical outcomes in patients with ESRD underwent spine surgeries, but no one discussed about which fusion method was optimal for this kind of patients. In this study, we compared the differences between lumbar posterolateral fusion (PLF) and lumbar interbody fusion (IBF) in uremic patients underwent instrumented lumbar surgeries.

**Methods:**

Between January 2005 and December 2017, ESRD patients under maintenance hemodialysis underwent posterior instrumented fusion for DLD were reviewed. A PLF group and an IBF group were identified. The demographic data was collected using their medical records. Clinical outcomes were evaluated by Oswestry Disability Index (ODI) and the visual analogue scale (VAS); radiographic results were assessed using final fusion rates. Any surgical or implant-related complication was documented.

**Results:**

A total of 34 patients (22 women and 12 men, mean age of 65.4 years) in PLF group and 45 patients (26 women and 19 men, mean age of 65.1 years) in IBF group were enrolled. Both groups had similar surgical levels. The operation time was longer (200.9 vs 178.3 min, *p* = 0.029) and the amount of blood loss was higher (780.0 vs 428.4 ml, *p* = 0.001) in the IBF group. The radiographic fusion rate was better in the PLF group but without significant difference (65.2% vs 58.8%, *p* = 0.356). Seven in the PLF group and ten in the IBF group developed surgical complications (20.5% vs. 22.2%, *p* = 0.788); three patients in the PLF group (8.8%) and five patients in the IBF group (11.1%) received revision surgeries because of implant-related or wound complications. Comparing to preoperative ODI and VAS, postoperative ODI and VAS obtained significant improvement in both groups.

**Conclusions:**

Successful fusion rates and clinical improvement (VAS, ODI) were similar in IBF and PLF group. Uremic patients underwent IBF for DLD had longer length of operation and higher operative blood loss than underwent PLF.

## Introduction

According to the US Renal Data System’s (USRDS) Annual Data Report, the incidence and prevalence of end-stage renal disease (ESRD) in Taiwan are the highest in the world [1]. More than 80,000 Taiwanese patients currently require dialysis, and that number is increasing [2]. Owing to advances in hemodialysis techniques, ESRD patients have a longer lifespan than previously reported. This implies that degeneration of the musculoskeletal system, such as that seen in degenerative lumbar diseases (DLD), becomes more symptomatic with age in patients with ESRD. DLD frequently requires patients to undergo surgical decompression and fusion. Lumbar posterolateral fusion (PLF) and lumbar interbody fusion (IBF) are the two main surgical techniques used for fusion in DLD. Instrumented PLF has been practiced for over three decades in DLD patients. Because anterior structures of the spine are not supported in PLF; therefore, a higher incidence of pseudarthrosis may occur. IBF with cage decompresses the neural tissue by increasing the foramen height, thereby providing anterior support of the spinal column and theoretically increasing the fusion rate. However, there is still debate as to which method is optimal for DLD [3].

With the increasing number of hemodialysis patients requiring surgeries for their DLD in our country, we are being consulted for surgical options with increasing frequency. In a literature review, only a few sporadic studies discussed surgical outcomes and their associated complications in ESRD patients who underwent spine surgeries [4, 5], but no reports have discussed which fusion method is optimal for patients of this description. A hypothesis was proposed that ESRD patients underwent lumbar instrumented surgeries might have more advantages by using the IBF method than using the PLF method. Therefore, we conducted this study to assess the outcomes in patients with hemodialysis dependence who have undergone instrumented lumbar surgeries and focused on comparisons between lumbar posterolateral fusion (PLF) and lumbar interbody fusion (IBF).

## Materials and methods

**Fig. 1:**
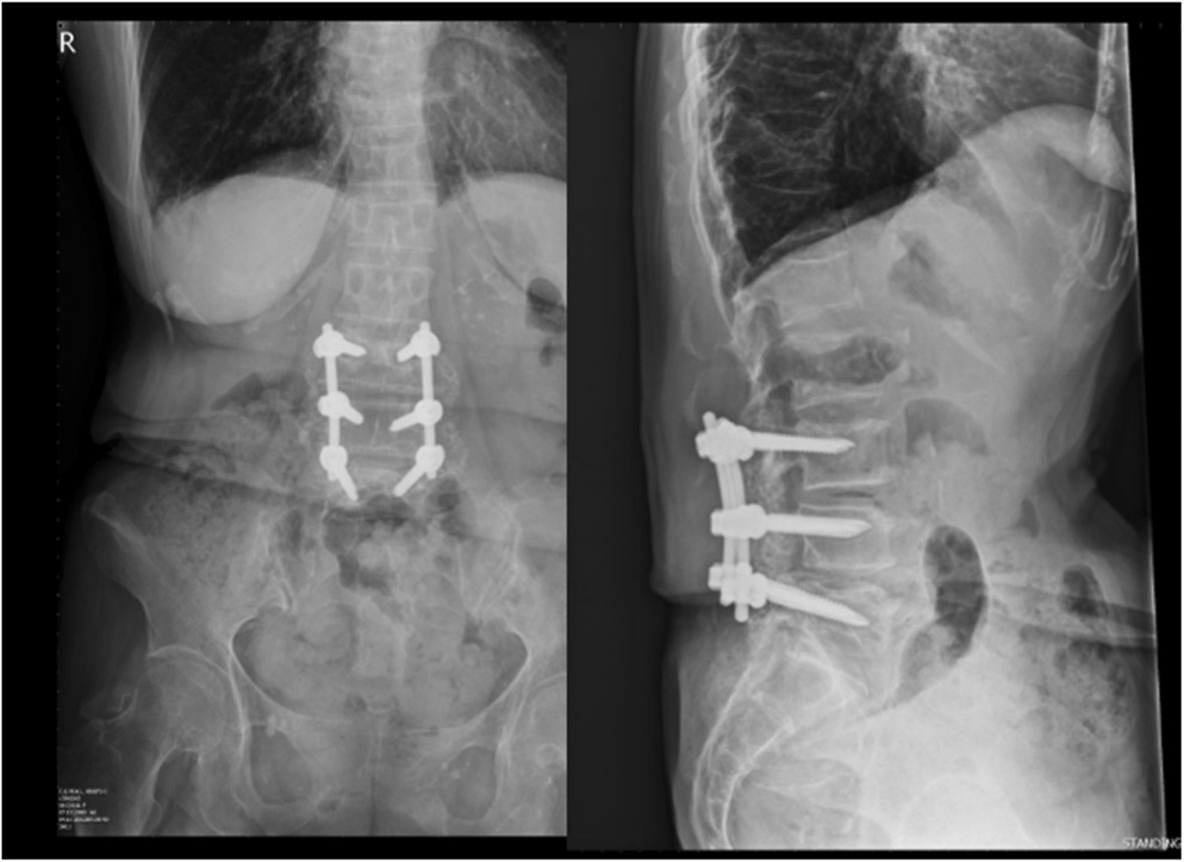
A case in the PLF group: L3–4-5 posterior instrumentation, decompression, and posterolateral fusion

**Fig. 2:**
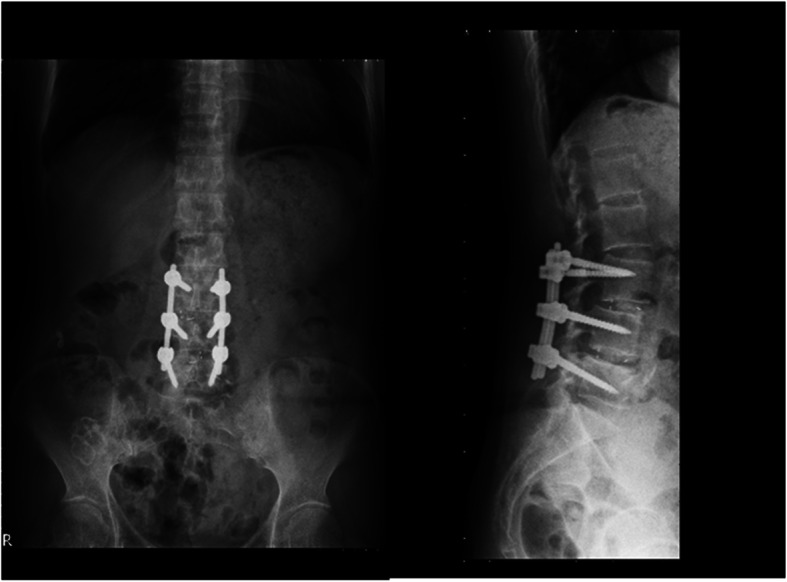
A case in the IBF group: L3–4-5 posterior instrumentation, decompression, and interbody fusion with cages

Patients receiving maintenance hemodialysis for ESRD who underwent posterior instrumented lumbar spinal surgery for DLD at Chang Gung Memorial Hospital between January 2005 and December 2017 were reviewed. The inclusion criteria were spondylolytic spondylolisthesis, degenerative lumbar spondylolisthesis, degenerative lumbar scoliosis, or degenerative lumbar kyphoscoliosis. The exclusion criteria included the presence of spine metastases, primary spinal cancer, spine trauma, spinal infection, or revision surgery. A total of 84 patients were included in this study. All patients who underwent posterior instrumented lumbar spinal surgery were divided into two groups based on the spinal fusion technique used: a PLF group (Fig. 1) and an IBF group (Fig. 2). These patients were followed up for at least two years after surgery. Surgical or implant-related complications were documented. Three patients were excluded from the PLF group: two died after the index surgery, one due to ischemic bowel disease during hospitalization and the other due to biliary tract infection one month after discharge, and the third died at follow-up after the index surgery. Two patients were excluded from the IBF group: one died due to choking-induced cardiac arrest three months after discharge, and the other died at follow-up after the index surgery. Therefore, this study included a PLF group of 34 patients and an IBF group of 45 patients. We will explain for all patients about higher success fusion rates were observed in IBF than PLF for general patients based on previous studies. Due to poor bone quality of ESRD patients, IBF will be suggested. However, the fee of interbody fusion cages is not covered by our National Health Insurance. Therefore, these two methods and their cost will be explained to the patients before surgery. The final fusion method will be determined after the surgeon discuss with the patient. All patients underwent decompression and posterior instrumentation. Certain patients received posterior lumbar interbody fusion or transforaminal interbody fusion with polyetheretherketone (PEEK) cages. The posterolateral spinal fusion technique was performed by adequate removal of the soft tissue at the fusion level, followed by decortication of the transverse process and placement of bone graft material along the sides of the fusion level to stimulate bone growth. The recorded surgical information included fusion level, operation time, and the amount of blood loss. Preoperative renal functions including blood urea nitrogen (BUN), creatinine (Cr), and estimated glomerular filtration rate (eGFR) were recorded. The severity of renal impairment was classified into five stages using Kidney Disease Outcome Quality Initiative (KDOQI) guidelines [6]. All comorbidities of patients, such as diabetes mellitus, hypertension, coronary artery disease, cerebral vascular accident, and cancer were documented. Each patient’s comorbidities were weighted using the Charlson Comorbidity Index (CCI) [7].

Clinical outcomes were evaluated using the visual analog scale (VAS) and Oswestry Disability Index (ODI). The VAS was measured before the operation and the most recent clinical visit. ODI was acquired using a retrospective survey administered by phone contact with patients. Radiographic evaluations were performed at the most recent clinical visit to check the stability of the implants and the solidity of the fusion mass. The fusion rate of the PLF group was calculated for each segment. For example, there are 2 segments in L4–5 posterolateral fusion and 4 segments in L3–L5 posterolateral fusion. The solid fusion of each segment was calculated separately. Interbody fusion was graded using the method described by Brantigan et al. as modified to describe the Fraser definition of locked pseudarthrosis (BSF scale) [8].

Statistical analysis was performed using the SPSS statistical software package. Continuous variables were compared between the two groups using the Mann-Whitney U test. Categorical variables were compared using the chi-squared test. A dependent Student’s t-test was used for comparisons between preoperative and postoperative VAS and ODI scores. A two-tailed value of *p* < 0.05 was considered statistically significant.

## Results

A total of 34 patients (22 women and 12 men; mean age, 65.4 years) were enrolled in the PLF group, and a total of 45 patients (26 women and 19 men; mean age, 65.1 years) were enrolled in the IBF group. There were no significant differences in the mean BUN (PLF: 47.3 vs IBF: 52.7 mg/dL, p = 0.146), Cr (PLF: 6.7 vs IBF: 6.9 mg/dL, p = 0.494), and eGFR (PLF: 7.4 vs IBF: 7.1 mL/min/1.73m^2^, p = 0.642) between the two groups. According to KDOQI guidelines, all patients in these two groups were classified as chronic kidney disease stage 5. There were no significant differences in age, gender distribution, or surgical levels between the groups. The mean CCI of the PLF group was 3.7 ± 1.2, and the mean CCI of the IBF group was 3.5 ± 1.5 (*p* = 0.577). The mean operation time was longer (210.9 vs. 178.3 min, *p* = 0.029) in the IBF group, and the amount of blood loss was higher (780.0 vs. 428.4 mL, *p* = 0.001). Table 1 compares the two groups in terms of the patients’ demographic data. All patients who underwent radiographic imaging for at least 12 months postoperatively were classified as solid fusion or inadequate fusion based on each segment in the PLF group, while the IBF group were classified as healed interbody fusion or not healed. The radiographic fusion rate was better in the PLF group, although the difference was not significant (65.2% vs. 58.8%, *p* = 0.356). All complications were recorded and divided into surgical or medical complications. Seven patients in the PLF group developed surgical complications (20.5%), including screw malposition (two cases), implant loosening (three cases), and wound dehiscence (two cases). Two patients in the PLF group developed medical complications (5.8%), including postoperative AV graft failure and lower gastrointestinal bleeding. Ten patients in the IBF group developed surgical complications (22.2%), including cage posterior migration (two cases), cage subsidence (four cases), spondylitis around the cage (one case), implant loosening (two cases), and wound dehiscence (one case). Three patients in the IBF group developed medical complications (6.6%), including postoperative hyperkalemia and AV graft failure. Three patients in the PLF group (8.8%) and five patients in the IBF group (11.1%) received revision surgeries because of implant-related or wound complications. There were no statistically significant differences in surgical and medical complications between the PLF and IBF groups (Table 2). The VAS was measured before the operation and before the latest clinic visit. The preoperative and postoperative mean VAS scores for back pain were 4.9 ± 1.4 and 2.3 ± 2.3 in the PLF group, while the respective scores were 5.0 ± 1.5 and 2.1 ± 2.1 in the IBF group. The preoperative and postoperative mean VAS scores for leg pain were 6.2 ± 1.9, 3.3 ± 2.3 in the PLF group, and the corresponding scores were 6.5 ± 2.1 and 3.5 ± 2.1) in the IBF group. The preoperative and postoperative mean ODI were lower in the PLF group (38.5 ± 14.2, 22.0 ± 12.1) than in the IBF group (39.3 ± 18.0, 23.9 ± 13.2). When compared the preoperative and postoperative ODI and VAS scores, significant improvement was observed in both groups. However, no significant differences were observed between the groups (Table 3).

**Table 1:** Patient Demographic Data (PLF vs. IBF)

Characteristic	PLF (*N* = 34)	IBF (*N* = 45)	*P* values
Age (years)	65.4 ± 8.4	65.1 ± 7.1	0.729
Gender			
Female	22	26	0.436
Male	12	19	
Surgical segements			
One-sgemnt	15	14	
Two-segment	15	21	
Three-segment	1	7	0.557
Four segment	3	3	
Operation time (min)	178.4 ± 52.0	210.9 ± 50.1	0.029
Blood loss (c.c.)	428.4 ± 201.5	780.0 ± 306.7	0.001
BUN (mg/dL)	52.7 ± 17.8	47.3 ± 15.7	0.146
Cr (mg/dL)	6.9 ± 2.3	6.7 ± 1.9	0.494
eGFR (mL/min/1.73m^2^)	7.1 ± 3.2	7.4 ± 2.2	0.642
CCI	3.7 ± 1.2	3.5 ± 1.5	0.577

*PLF* posterolateral fusion, *IBF* interbody fusion, *BUN* blood urea nitrogen, *Cr* creatinine, *eGFR* estimated glomerular filtration rate, *CCI* Charlson Comorbidity Index

**Table 2:** Surgical and medical complications (PLF vs. IBF)

Characteristic	PLF (*N* = 34)	IBF (*N* = 45)	*P* values
Surgical (number, %)	7 (20.5%)	10 (22.2%)	0.562
screw malposition	2	–	
implant loosening	3	2	
wound dehiscence	2	1	
cage migration	–	2	
cage subsidence	–	4	
spondylitis	–	1	
Medical (number, %)	2 (5.8%)	3 (6.6%)	0.724
AV graft failure	1	1	
Hyperkalemia	–	2	
Lower gastrointestinal bleeding	1	–	

*PLF* posterolateral fusion, *IBF* interbody fusion, *AV* arteriovenous

**Table 3:** **Clinical outcomes** (**ODI and VAS**) **(PLF vs. IBF)**

Characteristic	PLF (*N* = 34)	IBF (*N* = 45)	*p* values
VAS (back pain)			
preoperative	4.9 ± 1.4	5.0 ± 1.5	0.320
final	2.3 ± 2.3	2.1 ± 2.1	0.316
preoperative vs final	*p* < 0.001	*p* < 0.001	
VAS (leg pain)			
preoperative	6.3 ± 1.9	6.5 ± 2.1	0.452
final	3.3 ± 2.3	3.5 ± 2.1	0.561
preoperative vs final	*p* < 0.001	*p* < 0.001	
ODI			
preoperative	38.5 ± 14.2	39.3 ± 18.0	0.216
final	22.0 ± 12.1	23.9 ± 13.2	0.225
Preoperative vs final	*p* < 0.001	*p* < 0.001	

*VAS* visual analog scale, *ODI* Oswestry Disability Index, *PLF* posterolateral fusion, *IBF* interbody fusion

## Discussion

Surgery for patients with ESRD is challenging for surgeons. In a literature review, surgical complications, such as cardiovascular diseases, volume disturbance, coagulopathy, metabolic acidosis, and electrolyte imbalance were observed more frequently in ESRD patients who had undergone major surgery [9, 10].

Gajdos et al. followed 1506 ESRD patients who had undergone general surgery between 2005 and 2008. They reported that dialysis patients had a significantly greater rate of both 30-day overall complications (28.6% vs. 10.7%, *p* < 0.001) and unplanned return to the operating room (18.5% vs. 4.9%, p < 0.001) than did non-dialysis patients [11]. Surgical outcomes of dialysis patients who underwent orthopedic surgery were also poor, especially in trauma cases. Patients with ESRD experienced mortality rates of up to 50% at one year following hip fracture. High infection or sepsis rates have also been reported [12–14]. In another study, Ackland et al. reported a series of 142 patients with chronic kidney disease (CKD) (stages 3 through 5) undergoing elective primary and revision total hip arthroplasty (THA) and total knee arthroplasty (TKA). The authors observed a two-fold risk of surgical complications, such as pulmonary, infectious, cardiovascular, and gastrointestinal complications, as compared with those of normal patients [15].

Eric Nyam et al. reported high surgical risk and complications in patients with ESRD who underwent spinal surgery. Their series report included 4109 participants with ESRD and 8218 patients without ESRD, all of whom were undergoing spinal surgery. The authors observed comparatively poorer outcomes for the ESRD patients: ESRD patients who underwent spinal surgery presented significantly greater in-hospital mortality than did patients without ESRD (10.17% vs. 1.39%, *P* < 0.0001). Moreover, different spinal surgery methods also influence on ESRD patients’ in-hospital mortality rates: operations on spinal cords and spinal canal structures had the greatest hospital mortality (14.87%) compared with spinal fusion (3.46%), excision, or destruction of intervertebral disc (3.01%) [4]. Decompression and instrumented spinal fusion are frequently used for the surgical treatment of DLD. Lumbar posterolateral fusion (PLF) and lumbar interbody fusion (IBF) are the two main techniques of instrumented spinal fusion. Both of these have been extensively studied in prior reports. McAnany et al. conducted a systematic review of 865 articles that revealed no significant differences in clinical outcomes (VAS, ODI), surgical information (operation time, estimated blood loss), complication rate, or fusion rate between the two groups [16].

However, no study appears to have discussed which fusion method is optimal for patients with ESRD. Using the traditional open method, high blood loss is common in instrumented lumbar surgery because the spine is a rich blood supply area [17]. Coagulopathy or even disseminated intravascular coagulation (DIC) may result in significant blood loss, which is fatal and may cause postoperative epidural hematoma or increase the risk of infection. According to a previous study, coagulation abnormalities were observed in 42.9% of patients with ESRD [18]. The mechanism of ESRD coagulopathy is that uremic toxins inhibit normal platelet function and platelet–vessel wall interactions [19]. This is why ESRD patients with hemodialysis dependence can experience greater blood loss during instrumented lumbar surgery. In our study, the amount of blood loss was significantly higher (780.0 vs 428.4 ml, *p* = 0.001) in the IBF group than in the PLF group. Since epidural bleeding is common during IBF surgery, these findings suggest that IBF could aggravate blood loss in ESRD patients undergoing instrumented lumbar surgeries.

The mean operation time was longer (210.9 vs. 178.3 min, *p* = 0.029) in the IBF group than in the PLF group in the current study, meaning that the patients in the IBF group were anesthetized for longer. General anesthesia is required for patients undergoing instrumented lumbar surgery; however, general anesthesia can be very difficult for anesthesiologists to administer to patients with ESRD. Hemodynamic instability is common in patients with ESRD following the induction of general anesthesia [20]. Hence, anesthesiologists must evaluate each patient’s fluid status consistently and adjust fluid therapy carefully; the longer the time under anesthesia, the greater the surgical risk. In this study, patients with ESRD who underwent interbody fusion for lumbar surgery required a longer operation time and had a higher incidence of complications. The surgeon should consider the shortcomings of the IBF technique in these patients before surgery.

The radiographic outcome in our study was assessed using the fusion rate. In a literature review, the fusion success rate in general patients was approximately 84–90% when using PLF and 90–95% when using IBF [21, 22]. Generally, the fusion success rate is slightly higher in IBF than in PLF in general populations. However, no study on the success rate of fusion in patients with ESRD appears to exist. Based on our results, the fusion success rate was better in the PLF group than in the IBF group, although the difference was not significant (65.2% vs. 58.8%, *p* = 0.356). When compared to general patients, the fusion success rate was significantly lower in ESRD patients. Patients with ESRD usually have osteoporosis and exhibit several metabolic and hormonal abnormalities, including decreased renal synthesis of 1,25(OH)2D3, hyperphosphatemia, hypocalcemia, increased secretion of PTH, chronic metabolic acidosis, and, more recently, 25(OH) vitamin D deficiency, which may affect bone growth and remodeling processes [23]. According to our study, the rate of fusion success was lower in the IBF group than in the PLF group, while the opposite was true in non-ESRD patients. We believe that the poor result in the IBF group was attributed to poor bone quality in the ESRD patients. In the IBF group, we observed two cases with posterior cage migration and four cases with cage subsidence. Based on a previous study, osteoporosis is an important risk factor for cage migration or subsidence [24].

Regarding surgical and medical complications of ESRD patients after undergoing instrumented spinal fusion, Puvanesarajah et al. reported that patients with late-stage renal disease that had undergone1–2 level posterolateral lumbar fusion had 1.6 times higher risk of experiencing a major medical complication within 3 months of surgery and 2.8 times increased risk of 1-year mortality when compared with patients without renal disease [5]. Our results show that both groups had high surgical complications (20.5% vs. 22.2%), including implant loosening and wound dehiscence, as well as medical complications (5.8% vs. 6.6%), including electrolyte imbalance and AV graft failure. However, there were no statistically significant differences in surgical and medical complications between the PLF and IBF groups.

In a literature review, there was no significant difference in clinical outcomes between PLF and IBF for DLD [16]. In our study, patients with ESRD underwent PLF or IBF, and both groups showed significant improvements in ODI and VAS scores compared to baseline, but there were no significant differences between the groups.

Our study does, however, have some limitations. First, the surgeons chose the surgical method at their own discretion, thus potentially influencing the results by selection bias. Second, radiographic fusion and instrumentation failure were not evaluated by dynamic radiographs (flexion-extension view) or computed tomographic assessment, which might decrease accuracy. Third, the DLD of the current study included degenerative spondylolisthesis, degenerative lumbar scoliosis, degenerative lumbar kyphosis, and isthmic spondylolisthesis. These heterogeneous cohorts might decrease the generalizability to the study population and interfere with the results.

## Conclusions

ESRD patients who underwent IBF for DLD had a longer duration of operation and higher operative blood loss than those who underwent PLF. It appeared that IBF did not provide any advantages over PLF in ESRD patients who underwent surgeries for their DLD.

## Data Availability

All the necessary information is contained in the manuscript. The datasets used and/or analyzed during the current study are available from the corresponding author on reasonable request. Each participant’s raw data is only available in hospital archive. Therefore digital availability of each patient’s data is limited.
